# Prevalence, types and severity of medication errors associated with the use of automated medication use systems in ambulatory and institutionalized care settings: A systematic review protocol

**DOI:** 10.1371/journal.pone.0260992

**Published:** 2021-12-03

**Authors:** Kazeem Babatunde Yusuff, Mariam Mustafa, Najla Hezam Al-Qahtani

**Affiliations:** Department of Clinical Pharmacy and Practice, College of Pharmacy, QU Health, Qatar University, Doha, Qatar; University of Bern: Universitat Bern, SWITZERLAND

## Abstract

The use of automated systems within the medication use process has significantly reduce the occurrence of medication errors and the associated clinical and financial burden. However, automated systems lull into a false sense of security and increase the risk of medication errors that are often associated with socio-technical interactions, automation bias, workarounds and overrides. The objective of the systematic review is to determine the prevalence, types and severity of medication errors that are associated the use of automated systems in ambulatory and institutionalized care settings. The search strategy will be guided by PRISMA framework. Selected databases and relevant gray literature were searched and screening was done independently by two researchers between 01 April and 29 June 2021. These covered all relevant articles published from the inception of the use of automation in the medication use process (2000) until 2020. De-duplication and screening of all studies were done independently by two researchers with a clear inclusion / exclusion criteria. Data extraction and synthesis are currently on going (started on 06 July 2021) and being conducted independently but the validity and completeness of the processes will be confirmed by the third researcher. The Cochrane Risk of Bias tool and the Hoy et al’s quality assessment checklist will be used for the assessment of methodological bias while the Grading of Recommendations Assessment, Development, and Evaluation (GRADE) system will be used for the quality of evidence assessment. Detailed qualitative synthesis of key findings will be done with thematic and descriptive analyses. If the number and types of included studies permit, fixed or random effect model meta-analysis will be conducted based on the degree of homogeneity in the sampling frame used in the included studies. Heterogeneity will be assessed with I^2^ statistics and I^2^ > 50% will be considered a high statistical heterogeneity. The systematic review may provide new perspective especially from developing settings about the prevalence, types and severity of medication errors associated with the use of automated systems at all the stages of medication use process, and in all categories of patients. This may add to global knowledge in the research area. **Systematic review registration**: The systematic review was registered and published by PROSPERO (CRD42020212900).

## Introduction

Appropriate use of rationally prescribed medications is critical to the achievement of optimal outcomes, but this is predicated on an error-free medication use process that ensures that medications are used effectively and safely, and their movement from one stage to the other fully tracked and documented [1]. However, the medication use process is in reality complex and involves multiple stages and professionals with different background, and this is associated with an increased risk of medication errors and adverse drug events (ADEs) [2,3]. Bates et al. (1995) reported that 56% of all preventable ADEs occur at the prescribing phase followed by administration phase (34%), transcription phase (6%) and dispensing phase (4%) [4]. Medication errors are preventable events that may occur within the medication use process and cause or lead to inappropriate medication use or patient harm [5]. Medication errors are responsible for thousands of avoidable deaths, temporary or permanent harm and billions of dollars in financial loss [6,7]. However, the use of automated systems at the various stages of the medication use process have significantly reduce the occurrence of medication errors and the associated clinical and financial burden [8–11]. Automated systems such as electronic medical records, computerized physician order entry system, clinical decision support system, automated / robotic dispensing systems and bar code administration system have all contributed to significant reduction in medication errors and patient harms [12–17]. A systematic review of the effectiveness of automated systems in outpatients and community settings showed a 37% reduction in medication errors, increased productivity and reduced patient waiting and prescription filling time [18]. In addition, Sinnemaki et al 2013 in a systematic review of automated dose dispensing service in primary care settings showed increased accuracy of medication administration and appropriate use [19]. Furthermore, automated systems have also been reported to reduce workload, improve efficiency and reduce job-related stress [20]. However, automated systems are not error-proof as the observed improvement in medication use and patient safety associated with their use may lull healthcare professionals into a false sense of security from the occurrence of medication errors. This is because automated systems require a level of human interventions to function optimally, and hence the potential for errors exist. Indeed, the phenomenon of socio-technical interactions has been identified as a major factor underlining the risk of errors with automated system as the attitude of users and acceptance of automation and its appropriate use are critical to prevention of errors [21,22]. Furthermore, the automation of the various stages of the medication use process has thrown up new phenomenon such as automation bias, automation complacency, workarounds and overrides; and these have been identified as accounting for the rising prevalence of medications errors associated with the use of automated systems [23–25]. Furthermore, the complexity of the tasks or processes that are not supported or covered by automated systems, and which require human intervention have been identified as sources of errors including omission, wrong timing and medication mishandling [26–28]. Current published evidence about automated systems-related medication errors is relatively modest, and mostly focused on medication errors encountered at specific single stage of the medication use process or in specific patient group or healthcare setting. Hence, this systematic review will provide a comprehensive review of evidence of the frequency, types and severity of the medications errors associated with the use of automated systems at all the stages in the medication use process for all category of patients in both developed and developing settings. Hence, findings from this systematic review may potentially provide new perspective especially from developing settings that may add significantly to global knowledge in the research area. The objective of the systematic review is to determine the prevalence, types and severity of medication errors that are associated with the use of automated systems for adults and children in ambulatory and institutionalized care settings.

## Methods

### Protocol

The protocol was developed with the Preferred Reporting Items in Systematic Reviews and Meta-analyses (PRISMA) statement and this will guide all the activities related to the systematic review [29] (S1 Checklist). The International Prospective Register of Systematic Reviews (PROSPERO) registered the protocol for the systematic review (CRD42020212900) on November 07, 2020. The registered protocol will be updated in PROSPERO should need for any relevant amendment arise.

### Eligibility criteria

The criteria for inclusion in the systematic review are as follow:

1. All eligible studies with observational, experimental and quasi-experimental including cross-sectional, case-control, nested case-control, cohort and randomized, non-randomized, controlled and uncontrolled studies respectively. Relevant systematic reviews published in the research area will be hand-sampled for cross-referencing.
2. All eligible studies focused on the prevalence, types and severity of medications errors associated with all automated systems used in the prescribing, dispensing and administration stages of the medication use process.
3. All eligible studies published in English Language starting from 2000 to 2020
4. All eligible studies that meet the inclusion criteria irrespective of geographic location, setting, gender, age or race.

### Exclusion criteria

We will exclude case report and case series, narrative reviews, abstracts, personal opinions, commentaries, and conference reports. In addition, all studies with qualitative designs such as focus group discussions, semi-structured interviews and surveys, studies published in other languages beside English and studies published earlier than 2000 will be excluded. The year 2000 was chosen as the start for inclusion because this coincides with the introduction of automated systems into the medication use process. Animal studies and those involved with the use of automations for non-medicine use related purpose will also be excluded.

### Database search and literature retrieval

The search strategy was developed by the MM and NHA and reviewed for validity and comprehensiveness by the third author (KBY) because of his experience with conducting systematic reviews and developing search strategy. The database search was conducted between 01 April and 29 June 2021 and consisted of three stages including an initial search to identify relevant key words / search terms in the title and abstracts of all retrieved papers. This was followed by a more in-depth database search with the use of all the search terms and Boolean operators, truncation and wildcards in variety of combinations to ensure a detailed and complete search. Three electronic databases (PubMed, EMBASE and Cochrane library) will be searched by two authors (MM and NHA) to identify all studies that meet the inclusion criteria. The key search terms included were (Prevalence OR Types OR severity) AND (medication OR drug) errors AND (automated systems OR automation OR BCMA OR eMAR OR AMS OR ADM OR CPOE OR BCDD OR CDSS OR PDAS OR ADC OR PICS OR MCA) and Boolean operators were used to develop diverse combinations of search terms to maximize the effectiveness and efficiency. The third stage consisted of a thorough hand search of the reference lists of all full text articles that meet the inclusion criteria to identify other relevant eligible studies. Furthermore, grey literature including publications by the World Health Organization, Institute for Safe Medication Practices (ISMP), National Coordinating Council for Medication Error Reporting and Prevention (NCCMERP), Agency for Healthcare Research and Quality were searched. All identified studies were uploaded to EndNote to remove duplicates. Two of the authors (MM and NHA) independently screened the titles and abstracts to exclude ineligible studies, and the third author (KBY) checked the validity of the procedure used for screening.

### Data extraction

Data extraction is currently on going (Started on 06 July 2021). Full text articles will be examined independently by all the authors, and an agreement will be reached regarding inclusion based on the inclusion criteria. Any probable discrepancy during selection will be discussed and resolved with consensus. Data extraction will be done independently by two of the authors (MM & NHA) with a standard data extraction sheet that will developed for the review. The data extraction form will be pre-tested to ensure completeness of data capture. The process for data extraction will be checked for validity and completeness by the third author (KBY). The data to be extracted will include name of author(s), year of publication, country of origin, study objective (s), duration of study, study design, study setting, study participants and sample size, age, frequency, types and severity of medication error, method used for categorizing the type and severity of medication errors, stage of the medication use process, automated system involved, and factor (s) underlining the medication error associated with automated system.

### Assessment of risk of bias

The process for the assessment of the risk of bias will be standardized with a pre-assessment of a sample of the included studies by all the authors and the result will be discussed. All the three authors will do the risk of bias assessment independently and any probable disagreement will be discussed and resolved with consensus. This assessment will be done for all randomized controlled trials (if any) with the Cochrane Risk of Bias tool [30]. The Hoy et al’s quality assessment checklist will be used for the methodological quality assessment of all the included studies. This is a specific tool used for assessing the methodological quality of prevalence studies and classifies the risk of bias as low, moderate, or high. This tool is easy to use and has a high interrater overall agreement of 91% [31].

### Quality of evidence

The assessment of the quality of evidence for the outcome of interest (Prevalence, Types, and severity of medication errors associated with the use of automated systems) will be done independently by two of the authors. The Grading of Recommendations Assessment, Development, and Evaluation (GRADE) system will be used for the quality of evidence assessment [32], and this will range from very low, low, moderate to high.

### Data analysis and synthesis

Data analysis and evidence synthesis will include a fixed or random effect model meta-analysis depending on the degree of homogeneity in the sampling frame used in the included studies. Heterogeneity will be assessed with I^2^ statistics and I^2^ > 50% will be considered a high statistical heterogeneity. For the meta-analysis, a sensitivity analysis will be done, if the number of included studies for the pooled analysis permit, to determine impact of the risk of bias on the robustness of the quantitative findings. Furthermore, we plan to assess the probability of publication bias with the use funnel plots to assess the possibility of publication bias if 10 more eligible studies were included for the meta-analysis [33]. However, a high degree of heterogeneity due to varieties of differences in study designs and settings, study participants, automated systems, medication use stages have been reported in the research area [34]. In addition, we do not anticipate the availability of a sizeable number of randomized controlled trials due to ethical reasons that may not allow the use of the classical double blind randomized controlled trial design in the research area. In addition, we plan to conduct a detailed qualitative synthesis that is consistent with the stated objective for the systematic review. The qualitative synthesis will include an arrangement of the key findings from all the included full text studies to relevant themes and / or sub-themes based. In addition, key findings related to methodological and participant characteristics, automated systems and associated types and severity of medication errors will be presented in tabular and / or graphical forms, including any other data related to the stated objective for the systematic review. Furthermore, we plan conduct descriptive statistical analysis using frequency, mean, and standard deviation, median and interquartile range as necessary.

## Discussion

We did not apply to an Institutional Review Board for ethical approval because published data that cannot be traced to specific individuals will be used for the systematic review. The protocol for the systematic review will be focused on presenting a comprehensive review of published evidence about the frequency, types and severity of medication errors that encountered with the use of automated systems at all the stages of medication use process. In addition, the review will focus on all categories of patients in both ambulatory and hospitalized health care settings and in both developed and developing settings. Several systematic reviews exists that report the evidence of the impact of the use of automated systems in reducing the occurrence of medication errors and patient harms, improving productivity, efficiency and patient safety. However, the introduction of automation for the delivery of medications has thrown up new type of medications errors that are mostly related to automation bias and complacency that are underlined by varieties of factors such as the complexity of interactions between human and technology, organizational and system-related factors and overreliance, workarounds and overrides. Current published evidence about automated systems-related medication errors is focused on those encountered at specific stage of the medication use process or in specific patient groups or healthcare settings. Hence, the proposed systematic review will provide a more comprehensive synthesis of evidence of the frequency, types and severity of the medications errors associated with the use of automated systems at all the stages in the medication use process in both developed and developing settings. Hence, findings from this systematic review may provide information on appropriate points for interventions to improve the safety of the use of automated systems within the medication use process. In addition, the systematic review may potentially provide new perspective especially from developing settings that may add to global knowledge in the research area.

### Strengths and limitations of this study

- The proposed systematic review will provide new perspectives especially from developing settings that may add significantly to global knowledge in the research area.
- The search strategy, including the database search, duplicate screening, selection of eligible studies, and data extraction, and assessment of methodological quality will be done with strict adherence to PRISMA standards.
- Cochrane Risk of Bias tool and The Hoy et al’s quality assessment checklist will be used for the assessment of methodological bias while the Grading of Recommendations Assessment, Development, and Evaluation (GRADE) system will be used for the quality of evidence assessment.
- Fixed or random effect meta-analysis will be conducted based on the degree of homogeneity the sampling frame use in the studies in the included studies. Homogeneity will be assessed with I^2^ statistics and I^2^ > 50% will be considered a high statistical heterogeneity
- Despite the planned strict adherence to the PRISMA guide, probable interference due to inclusion of only English language articles, combinations of the search terms and the databases to be used remain a possibility.

## Supporting information

S1 Checklist PRISMA-P 2015 checklist. (DOCX)

## References

[1] Wolfe D, Yazdi F, Kanji S, Burry L, Beck A, Butler C, et al. Incidence, causes, and consequences of preventable adverse drug reactions occurring in in-patients: A systematic review of systematic reviews. PLoS ONE. 2018; 13(10):e0205426. doi: 10.1371/journal.pone.0205426 30308067 PMC6181371

[2] Allan EL, Barker KN. Fundamentals of medication error research. Am J Hosp Pharm. 1990; 47: 555–71. 2180287

[3] Lisby M, Nielsen LP, Mainz J. Errors in the medication process: frequency, type, and potential clinical consequences. Int J Qual Health Care 2005;17:15–22. doi: 10.1093/intqhc/mzi015 15668306

[4] Bates DW, Cullen DJ, Laird N, Petersen LA, Small SD, Servi D et al. Incidence of adverse drug events and potential adverse drug events in hospitalized adults: Implications for prevention. Journal of American Medical Association. 1995; 274: 29–34. 7791255

[5] Risør BW, Lisby M, and Sørensen J. An automated medication system reduces errors in the medication administration process: results from a Danish hospital study. Eur J Hosp Pharm 2016;23: 189–196. doi: 10.1136/ejhpharm-2015-000749 31156847 PMC6451540

[6] Institute of Medicine. Preventing Medication Errors. Washington, D.C.: National Academies Press; July 2006.

[7] Institute for Safe Medication Practices. The National Medication Errors Reporting Program (ISMP MERP). Available at: https://www.ismp.org/orderforms/reporterrortoISMP.asp. Accessed April 30, 2020.

[8] Roumeliotis N, Sniderman J, Adams-Webber T, Addo N, Anand V. et al. Effect of Electronic Prescribing Strategies on Medication Error and Harm in Hospital: A Systematic Review and Meta-analysis. J Gen Intern Med.2019;34(10):2210–2223. doi: 10.1007/s11606-019-05236-8 31396810 PMC6816608

[9] Risør BW, Lisby M, and Sørensen J. Complex automated medication systems reduce medication administration errors in a Danish acute medical unit. International Journal for Quality in Health Care. 2018; 30(6), 457–465. doi: 10.1093/intqhc/mzy042 29590354

[10] Li Q, Kirkendall ES, Hall ES, Ni Y, Lingren T, Kaiser M et al. Automated Detection of Medication Administration Errors in Neonatal Intensive Care. J Biomed Inform. 2015;57: 124–133. doi: 10.1016/j.jbi.2015.07.012 26190267 PMC4715992

[11] Risør BW, Lisby M, Sørensen J. Cost-Effectiveness Analysis of an Automated Medication System Implemented in a Danish Hospital Setting. Value in Health. 2017; 20: 886–893. doi: 10.1016/j.jval.2017.03.001 28712617

[12] Longhurst CA, Parast L, Sandborg CI, Widen E, Sullivan J, Hahn JS, Dawes CG, Sharek PJ. Decrease in hospital-wide mortality rate after implementation of a commercially sold computerized physician order entry system. Pediatrics. 2010; 126(1):14–21. doi: 10.1542/peds.2009-3271 20439590

[13] Chapuis C, Roustit M, Bal G, et al. Automated drug dispensing system reduces medication errors in an intensive care setting. Crit Care Med 2010;38:2275–81. doi: 10.1097/CCM.0b013e3181f8569b 20838333

[14] Cousein E, Mareville J, Lerooy A, et al. Effect of automated drug distribution systems on medication error rates in a short-stay geriatric unit. J Eval Clin Pract 2014;20:678–84. doi: 10.1111/jep.12202 24917185 PMC4524418

[15] Poon EG, Keohane CA, Yoon CS, et al. Effect of bar-code technology on the safety of medication administration. N Engl J Med 2010;362:1698–707. doi: 10.1056/NEJMsa0907115 20445181

[16] McKibbon KA, Lokker C, Handler SM, Dolovich LR, Holbrook AM, O’Reilly D, et al. The effectiveness of integrated health information technologies across the phases of medication management: a systematic review of randomized controlled trials. J Am Med Inform Assoc. 2012; 19(1):22–30. doi: 10.1136/amiajnl-2011-000304 21852412 PMC3240758

[17] Hemens BJ, Holbrook AM, Tonkin M, Mackay JA, Weise-Kelly L, Navarro T, et al. Computerized clinical decision support systems for drug prescribing and management: a decision-maker-researcher partnership systematic review. Implement Sci. 2011; 3:6:89. doi: 10.1186/1748-5908-6-89 21824383 PMC3179735

[18] Sng Y, Ong CK, Lai YF. Approaches to outpatient pharmacy automation: a systematic review. Eur J Hosp Pharm. 2018;0:1–6. doi: 10.1136/ejhpharm-2017-001424 31428324 PMC6684014

[19] Sinnemäki J, Sihvo S, Isojärvi J, et al. Automated dose dispensing service for primary healthcare patients: a systematic review. Syst Rev 2013;2:1 doi: 10.1186/2046-4053-2-1 23295105 PMC3598731

[20] James KL, Barlow D, Bithell A, et al. The impact of automation on pharmacy staff experience of workplace stressors. Int J Pharm Pract 2013;21:105–16 doi: 10.1111/j.2042-7174.2012.00231.x 23418779

[21] Redwood S, Rajakumar A, Hodson J, Coleman JJ. Does the implementation of an electronic prescribing system create unintended medication errors? A study of the sociotechnical context through the analysis of reported medication incidents. BMC Med Inform Decis Mak. 2011;11: 29. doi: 10.1186/1472-6947-11-29 21569397 PMC3116457

[22] Karsh BT, Holden RJ, Alper SJ, Or CK (2006) A human factors engineering paradigm for patient safety: designing to support the performance of the healthcare professional. Qual Saf Health Care. 2006;15(1): 59–65. doi: 10.1136/qshc.2005.015974 17142611 PMC2464866

[23] Koppel R, Wetterneck T, Telles JL, Karsh BT. Workarounds to barcode medication administration systems: their occurrences, causes, and threats to patient safety. J Am Med Inform Assoc. 2008;15: 408–423. doi: 10.1197/jamia.M2616 18436903 PMC2442264

[24] Magrabi F, Ong M, Runciman W, Coiera E. An analysis of computer-related patient safety incidents to inform the development of a classification. J Am Med Inform Assoc 2010;17:663e670. doi: 10.1136/jamia.2009.002444 20962128 PMC3000751

[25] Pennsylvania Patient Safety Reporting System (PA-PSRS). Problems Associated with Automated Dispensing Cabinets. Patient Safety Advisory. 2005; 2(3): 1–4.

[26] Van den Bemt PMLA, Robertz R, De Jong AL, Van Roon EN, Leufkens HG. Drug administration errors in an institution for individuals with intellectual disability: An observational study. J Intellect Disabil Res 2007;51:528–36. doi: 10.1111/j.1365-2788.2006.00919.x 17537166

[27] Van denbemt PLA, Idzinga JC, Robertz H, Kormelink DG, Pels N. Medication Administration Errors in Nursing Homes Using an Automated Medication Dispensing System J Am Med Inform Assoc.2009;16: 486–492 doi: 10.1197/jamia.M2959 19390109 PMC2705251

[28] Kathleen E Walsh, William G Adams, Howard Bauchner, Robert J Vinci, John B Chessare, Maureen R Cooper et al. Medication Errors Related to Computerized Order Entry for Children. Pediatrics. 2006 Nov;118(5):1872–9. doi: 10.1542/peds.2006-0810 17079557

[29] Moher D, Shamseer L, Clarke M, Ghersi D, Liberati A, Petticrew M, et al. Preferred reporting items for systematic review and meta-analysis protocols (PRISMA-P) 2015 statement. Syst Rev. 2015;4(1):1. doi: 10.1186/2046-4053-4-1 25554246 PMC4320440

[30] Higgins JP, Altman DG, Gotzsche PC, Juni P, Moher D, Oxman AD, et al. The Cochrane Collaboration’s tool for assessing risk of bias in randomised trials. BMJ (Clinical research ed). 2011;343:d5928. doi: 10.1136/bmj.d5928 22008217 PMC3196245

[31] Hoy D, Brooks P, Woolf A, Blyth F, March L, Bain C, et al. Assessing risk of bias in prevalence studies: modification of an existing tool and evidence of interrater agreement. Journal of Clinical Epidemiology. 2012;65(9):934–9. doi: 10.1016/j.jclinepi.2011.11.014 22742910

[32] GRADE Working Group. Grading of recommendations assessment, development and evaluation. 2017. doi: 10.1177/1356389017733210 30369828 PMC6187832

[33] Korb-Savoldelli V, Boussadi A, Durieux P, Sabatiera B. Prevalence of computerized physician order entry systems–related medication prescription errors: A systematic review. International Journal of Medical Informatics. 2018: 112–122. doi: 10.1016/j.ijmedinf.2017.12.022 29425622

[34] Bezuidenhout H, Wiysonge CS, Bentley JM. Risperidone for disruptive behaviour disorders in children with intellectual disabilities [Protocol]. Cochrane Database of Sys Rev 2012;(7):CD009988.

